# Evolutionary Selection for Arsenic Resistance: The Case of the Atacameños of the Andes Highlands

**DOI:** 10.1289/ehp.121-a31

**Published:** 2013-01-01

**Authors:** Wendee Nicole

**Affiliations:** Wendee Nicole, based in Houston, TX, has written for *Nature*, *Scientific American*, *National Wildlife*, and other magazines.

In many locations the contamination of drinking water with inorganic arsenic has occurred relatively recently in evolutionary time. But in the Andes highlands, people have consumed water containing arsenic for at least several thousand years due to pre-Columbian mining activities and arsenic-tinged natural reservoirs; Andean mummies dating back 7,000 years have high arsenic concentrations in their hair and internal organs. Some populations in the Andes, including the Atacameño people living in San Antonio de los Cobros (SAC), Argentina, carry a particular variant (haplotype) of the arsenic-metabolizing gene *AS3MT* that is associated with more efficient arsenic metabolism and perhaps lower health risks from exposure. A team of investigators studied whether arsenic exposure may have provided evolutionary selection pressure for increased resistance in this population [*EHP* 121(1):53–58; Schlebusch et al.].

The investigators compared variants of the *AS3MT* gene between 346 SAC Atacameños and members of several other indigenous populations, including 25 individuals from three Native American populations participating in the Human Genome Diversity Project (HGDP) and 97 individuals from five Peruvian populations with historically lower exposure to arsenic. The team also measured urinary arsenic in the SAC population to look for differences in how individuals with different *AS3MT* variants metabolized arsenic.

Humans metabolize inorganic arsenic by a series of methylation reactions, first into methylarsonic acid (MMA) and then into dimethylarsinic acid (DMA), both of which are excreted in urine. Wide variation exists in human arsenic metabolism, and individuals who metabolize arsenic more efficiently will excrete more DMA and less MMA. Scientists often use urine’s fraction of MMA—the more toxic metabolite—as a marker of a person’s potential susceptibility to the toxic effects of arsenic.

**Figure:**
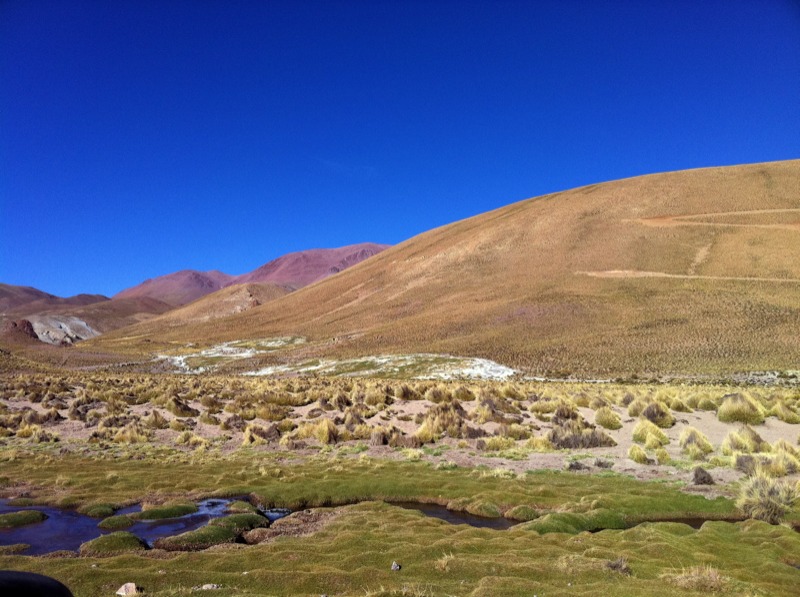
An arsenic-rich stream flows about 12 miles outside San Antonio de los Cobres. The Atacameño people have lived in this region for 11,000 years. © Karin Broberg Palmgren

Indigenous peoples of the Andes, including the SAC Atacameños, have been shown to excrete very low levels of MMA. This population contains a haplotype of the *AS3MT* gene that previous studies have associated with increased efficiency in arsenic methylation.

In the present study, the investigators found that 68.7% of SAC Atacameños carried the arsenic-protective *AS3MT* haplotype, compared with 50.5% of the Peruvians and 14.3% of the HGDP populations. Likewise, a haplotype that has *not* been associated with more efficient arsenic metabolism appeared in only 26% of the SAC population, compared with 67% of HGDP individuals and 40% of Peruvians.

There were no major differences between the SAC Atacameños and other indigenous populations when assessing genetic markers used to identify the degree to which populations are genetically distinct. This suggests that the higher prevalence of the protective *AS3MT* haplotype within the SAC individuals does not merely reflect general genetic differences between populations, but rather provides evidence for evolutionary pressure that led to arsenic-protective adaptation in the *AS3MT* gene.

At certain concentrations, arsenic is known to increase the risk of childhood morbidity and mortality, and adult cancer, diabetes, and cardiovascular disease. These new results suggest that arsenic exposure in this population may have historically reduced survival and reproduction rates enough that it led to a strong selection for individuals carrying arsenic-resistant haplotypes.

